# Stem Cell Factor Expression after Renal Ischemia Promotes Tubular Epithelial Survival

**DOI:** 10.1371/journal.pone.0014386

**Published:** 2010-12-21

**Authors:** Geurt Stokman, Ingrid Stroo, Nike Claessen, Gwendoline J. D. Teske, Jan J. Weening, Jaklien C. Leemans, Sandrine Florquin

**Affiliations:** Department of Pathology, Academic Medical Center, University of Amsterdam, Amsterdam, The Netherlands; University of Hong Kong, Hong Kong

## Abstract

**Background:**

Renal ischemia leads to apoptosis of tubular epithelial cells and results in decreased renal function. Tissue repair involves re-epithelialization of the tubular basement membrane. Survival of the tubular epithelium following ischemia is therefore important in the successful regeneration of renal tissue. The cytokine stem cell factor (SCF) has been shown to protect the tubular epithelium against apoptosis.

**Methodology/Principal Findings:**

In a mouse model for renal ischemia/reperfusion injury, we studied how expression of c-KIT on tubular epithelium and its ligand SCF protect cells against apoptosis. Administration of SCF specific antisense oligonucleotides significantly decreased specific staining of SCF following ischemia. Reduced SCF expression resulted in impaired renal function, increased tubular damage and increased tubular epithelial apoptosis, independent of inflammation. In an *in vitro* hypoxia model, stimulation of tubular epithelial cells with SCF activated survival signaling and decreased apoptosis.

**Conclusions/Significance:**

Our data indicate an important role for c-KIT and SCF in mediating tubular epithelial cell survival *via* an autocrine pathway.

## Introduction

One of the features of acute renal failure as induced by renal ischemia is the loss tubular epithelial cells (TEC) which significantly contributes to disruption of renal function. Therefore the development of new therapeutic interventions that prevents further loss of TEC caused by ischemia is essential to reduce kidney failure and to avoid the need for renal replacement therapy.

Recent studies demonstrate that the kidney can undergo effective repair following ischemia/reperfusion (I/R) injury. Distinct sources of TEC progenitors which are engaged in the re-epithelialization process have been described. Beside the contribution of bone marrow-derived stem cells [1], [2] and putative renal TEC stem cells [3] to kidney repair, the original hypothesis which states that viable TEC which have survived the ischemic insult start to proliferate and thereby generate new TEC that replace lost TEC, still stands [4], [5], [6].

The cytokine stem cell factor (SCF) and its receptor c-KIT are important in inducing cell differentiation, proliferation and survival in various cell types [7]. The receptor c-KIT is a tyrosine kinase receptor, belonging to the same subclass as platelet derived growth factor receptor. Its ligand SCF has to form a dimer to be able to induce signaling. Two splice variants of SCF have been reported in mice which differ in their expression of the 6^th^ exon [8]. This exon codes for an extracellular cleavage site, which is susceptible to proteolytic cleavage by proteases. Expression of the SCF variant containing exon 6 will produce a 45 kD membrane bound isoform, designated as Kit Ligand-1 (KL-1), whereby proteolytic cleavage will yield a 31 kD soluble form. Expression of the second SCF splice variant, lacking exon 6, results in a 32 kD membrane bound protein, KL-2. Although primarily found on cell membranes, shedding of KL-2 may still occur (reviewed in [9]). The expression ratio between the KL-1 and KL-2 isoforms of SCF varies significantly between various cell types [10].

SCF and c-KIT regulate diverse functions during hematopoiesis [11], gametogenesis [12] but also neural stem cell migration to the site of brain injury [13], [14], and melanocyte migration and survival [15]. Expression of c-KIT is upregulated or subject to gain-of-function mutations in several human neoplasms such as gastrointestinal stromal tumors [16], acute hematopoietic malignancies [17] and small cell lung cancer [18]. Expression of c-KIT occurs in distal nephrons of adult kidneys and in renal neoplasms [19], [20].

An important role for SCF and c-KIT has been described during nephrogenesis were a novel identified group of c-KIT positive progenitor cells may influence renal development[21]. In mouse models for acute renal failure, apoptosis following folic acid administration and I/R injury could be reduced by treatment with SCF [22]. However, the exact mechanism of SCF-mediated protection against apoptosis in I/R injury remains unclear. In this study we examined how SCF mediates survival of the tubular epithelium during I/R injury. Specific downregulation of SCF expression in the corticomedullary region of the kidney resulted in increased tubular damage and severely impaired renal function. We demonstrate that *in vitro* hypoxic conditions induce SCF expression and exposure to SCF promotes survival signaling via activation of c-KIT involving phosphorylation of Ser136 of Bad, leading to reduced caspase 3 activation. The SCF/c-KIT signaling route following ischemia provides a new opportunity to reduce TEC loss and to improve renal function after acute renal failure.

## Results

### Expression of c-KIT and SCF in the normal and ischemic kidney

**Figure 1:**
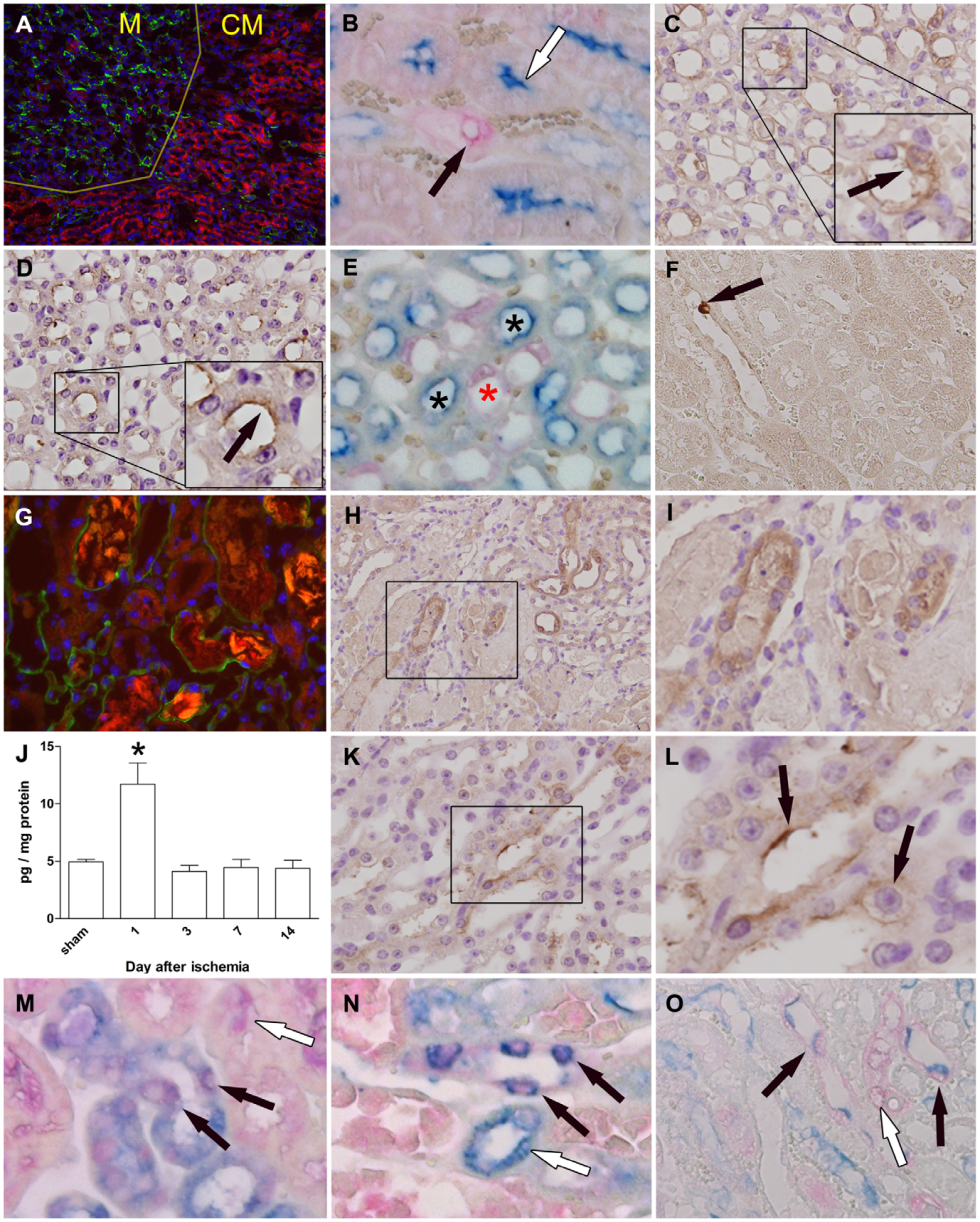
Expression of c-KIT and SCF in normal and ischemic kidneys. (A) Low magnification overview of the corticomedullary region (lower right, indicated as CM) and renal papilla (upper left, indicated as M) from a sham operated animal. Expression of c-KIT (green) is primarily localized to the distal nephrons located in the renal papilla. Tubules located in the corticomedullary region were stained for F actin (red) and show no clear c-KIT expression, whereas medullary rays extending from the papilla contain c-KIT positive cells. Nuclei (blue) were counterstained using DAPI. Original magnification: 10×. (B) Double immunostaining for CD10 (blue) and c-KIT (red) demonstrates that proximal tubules expressing CD10 located in the corticomedullary area do not express c-KIT (indicated by white arrow), whereas CD10 negative tubules may contain c-KIT expressing cells (indicated by black arrow). Original magnification: 40×. (C) Expression of c-KIT (brown) could be detected in tubules located in the renal papilla in sham operated animals. Nuclei were counterstained with hematoxylin (blue); original magnification: 40×. (D) SCF expression (brown) was located in the distal nephrons of sham operated animals and located at the luminal membrane of cells (indicated by arrow). Nuclei were counterstained with hematoxylin (blue); original magnification: 40×. (E) Double immunostaining for c-KIT (red) and SCF (blue) of a normal mouse kidney shows a distinct staining pattern in which tubule segments containing c-KIT expressing cells (marked with black asterisk) are separate from those that stain for SCF only (marked with red asterisk). (F) Single cell expressing SCF (brown, indicated by arrow) in the corticomedulary area demonstrates that SCF expression in the normal kidney is mostly absent. Original magnification: 20×. (G) High magnification view of tubules located in the corticomedullary area of the kidney 1 day after ischemia. F actin (red) staining shows deposition of brush border material in the lumen of damaged tubules. Epithelial cells of tubules now express c-KIT (green). Nuclear counterstaining (blue) using DAPI, original magnification: 40×. (H) Expression of c-KIT (brown) was equally confirmed on kidney tissue from animals one day after renal I/R injury. C-KIT was expressed by tubules in the corticomedullary region which contain cast deposition. Nuclei were counterstained with hematoxylin (blue); original magnification: 20×. (I) Magnification of outlined area in figure 1H, demonstrating expression of c-KIT by cells of damaged tubule segments. (J) Analysis of whole kidney samples by ELISA demonstrated a significant increase in renal SCF one day after ischemia, compared to renal SCF levels in sham operated animals and those at later time points (*P = 0.003). Data are expressed as mean±SEM. (K) One day after ischemia SCF (brown) was also present in damaged tubules located in the corticomedullary area. Nuclei were counterstained with hematoxylin (blue); original magnification: 40×. (L) Magnification of outlined area in figure 1K, demonstrating membrane staining for SCF (indicated by arrows). (M) and (N) Double immunostaining for c-KIT (red) and SCF (blue) at day 1 after ischemia demonstrating c-KIT and SCF specific staining in the same tubule segment (indicated by black arrows) in the corticomedullary area of the kidney. (M) C-KIT expression in the absence of SCF was also observed (white arrow), as well as (N) SCF expression in a tubule segment without cells expressing c-KIT (white arrow). Original magnification: 40×. (O) Double immunostaining for c-KIT (red) and CD10 (blue) at day 1 after ischemia demonstrates co-expression of c-KIT and CD10 (indicated by black arrows) and tubular epithelium that expresses c-KIT only (indicated by white arrows). Original magnification: 40×.

In normal adult human and mouse kidneys, expression of c-KIT has been reported to be limited to the distal nephrons [19], [20], [22]. In agreement with these findings we detected c-KIT expression on renal tissue sections of adult mice in the papilla and medullary rays, but not by tubules located in the corticomedullary area (figure 1A). Cells from tubules located in the corticomedullary expressing CD10 did not express c-KIT in normal mouse kidney (figure 1B), but c-KIT expression was evident in the distal nephrons of the normal kidney (figure 1C). Immunostaining of tissue sections from sham operated animals demonstrated SCF expression to be localized at the distal nephrons in the renal papilla (figure 1D and E) but virtually absent from the corticomedullary area (figure 1F). In contrast, one day after induction of renal ischemia, c-KIT positive cells were also detected in damaged tubules in the corticomedullary region of the kidney (figure 1G). The pattern of c-KIT staining was not homogenously distributed among the epithelial cells that were present in the tubule, but rather showed a mosaic appearance (figure 1H, I, M, N). Only very few c-KIT positive cells were detected in the interstitium (not shown). SCF concentrations were measured by ELISA in renal homogenates; the total concentration of SCF was significantly higher at the first day after ischemia only, compared to sham operated animals and later time points after ischemia (figure 1J). One day after ischemia, SCF expression was also detected at the apical side of tubules located in the corticomedullary area (figure 1K and L). We confirmed that c-KIT and SCF were present in the same tubule by performing double immunostainings (figure 1M and N). This suggests that SCF binding to and subsequent activation of c-KIT may occur *in vivo* during I/R injury. Following ischemia, expression of c-KIT was observed for tubular epithelial cells expressing CD10, identifying this population as of proximal tubular epithelium origin (figure 1O).

**Figure 2:**
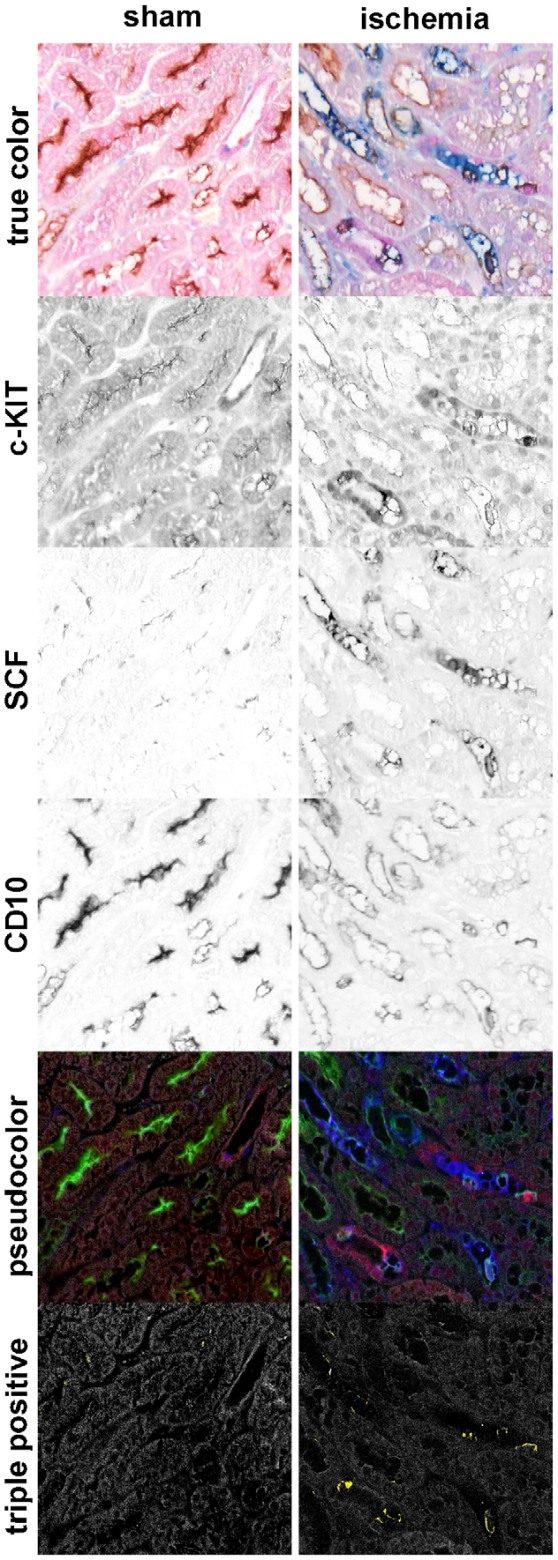
Spectral image analysis of triple immunostaining. Tissue sections were stained for c-KIT (red), SCF (blue), CD10 (brown) and counterstained with eosin (pink). Next, true color stainings were unmixed for each chromogen using spectral imaging (black and white images, eosin is not shown). Pseudocolors were assigned to c-KIT (red), SCF (blue), CD10 (green) and eosin (grey). Co-localization is presented in yellow. Left panel: sham-operated kidney, right panel: kidney at day 1 after ischemia. Original magnification: 20×.

Combined immunostainings for CD10, SCF and c-KIT were analyzed using spectral imaging to assess both co-expression by cells or co-localization per tubule (figure 2). In sham-operated animals, SCF and c-KIT specific staining was absent in tubules expressing CD10. During I/R injury, we detected both SCF single positive and SCF-c-KIT double positive tubules stained with CD10. These findings demonstrate that SCF and c-KIT expressing cells are present in proximal tubules.

### Decreased tubular SCF expression severely impairs renal function after ischemia

**Figure 3:**
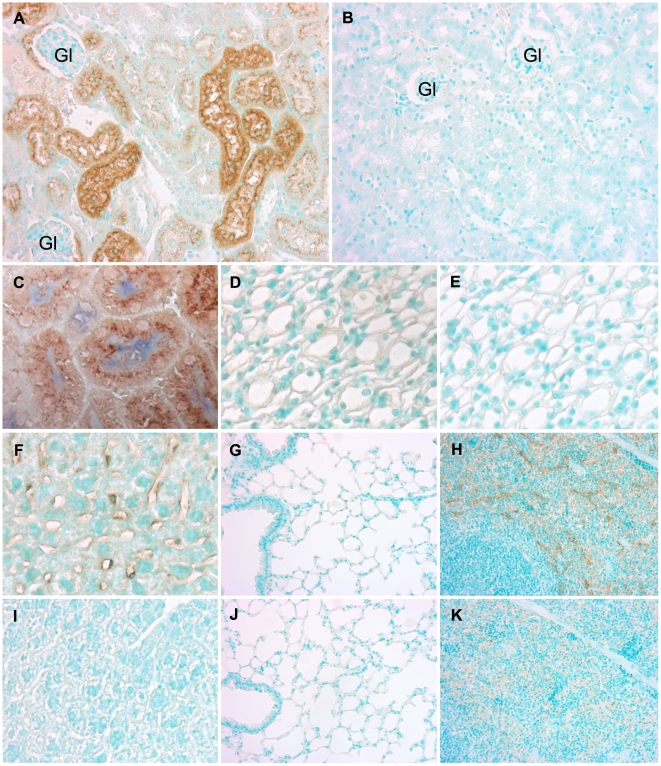
Tissue distribution of ODN. Distribution of FITC-labeled ODN was examined after two intraperitoneal administrations given with a 24 hour interval. Tissue was collected at 5 hours after the last administration. FITC was stained (brown) using a specific antibody showing (A) extensive uptake of FITC-labeled ODN by most tubule segments in the corticomedullary area and uptake by the parietal epithelium of the glomerulus (marked Gl) but (D) no significant uptake of ODN by the tubules located in the renal papilla. (C) CD10 expression (blue) by tubular epithelium co-stained for FITC (brown) demonstrates uptake of ODN by proximal tubules. (F) and (H) uptake of ODN by cells in liver and spleen and (G) no uptake of FITC-labeled ODN by lung tissue. Sections of (B) renal corticomedullary area, (E) renal papilla, (I) liver, (J) lung and (K) spleen labeled with secondary antibody only. Nuclei were counterstained with methyl green. Original magnification of all images: 40×.

We have previously demonstrated that antisense oligonucleotides (ASON) can effectively down regulate translation of proteins to which the ASON are targeted in tubular epithelial cells following intraperitoneal administration [23], [24]. To analyze the *in vivo* distribution and identify the nephron segments that take up oligonucleotides (ODN), we administered fluorescein isothiocyanate (FITC) labeled ODN twice with an interval of 24 hours and examined FITC-ODN uptake in kidney, liver, lung and spleen tissue 5 hours after the last administration. Uptake was detected immunohistochemically using a FITC-specific antibody. We found that FITC was present in most tubule segments located in the corticomedullary area, although the intensity of the staining varied per tubule (figure 3A). In addition, glomerular parietal epithelial cells also showed FITC-ODN uptake. Double staining for FITC-ODN and CD10 demonstrated the capability of proximal tubules in the corticomedullary area to take up ASON (figure 3C). In contrast, no uptake of ODN was found in tubules located in the renal papilla (figure 3D). Beside the kidney, we also found that FITC-ODN were taken up by cells in the liver (figure 3F) and the spleen (figure 3H) but not in the lung (figure 3G). Since expression of SCF in the ischemic kidney occurred in tubules located in the corticomedullary area and coincides with the emergence of c-KIT positive cells in tubules, we hypothesized that disruption of the SCF/c-KIT signaling pathway by administrating SCF-specific ASON may have implications for the epithelial response to ischemic injury.

**Figure 4:**
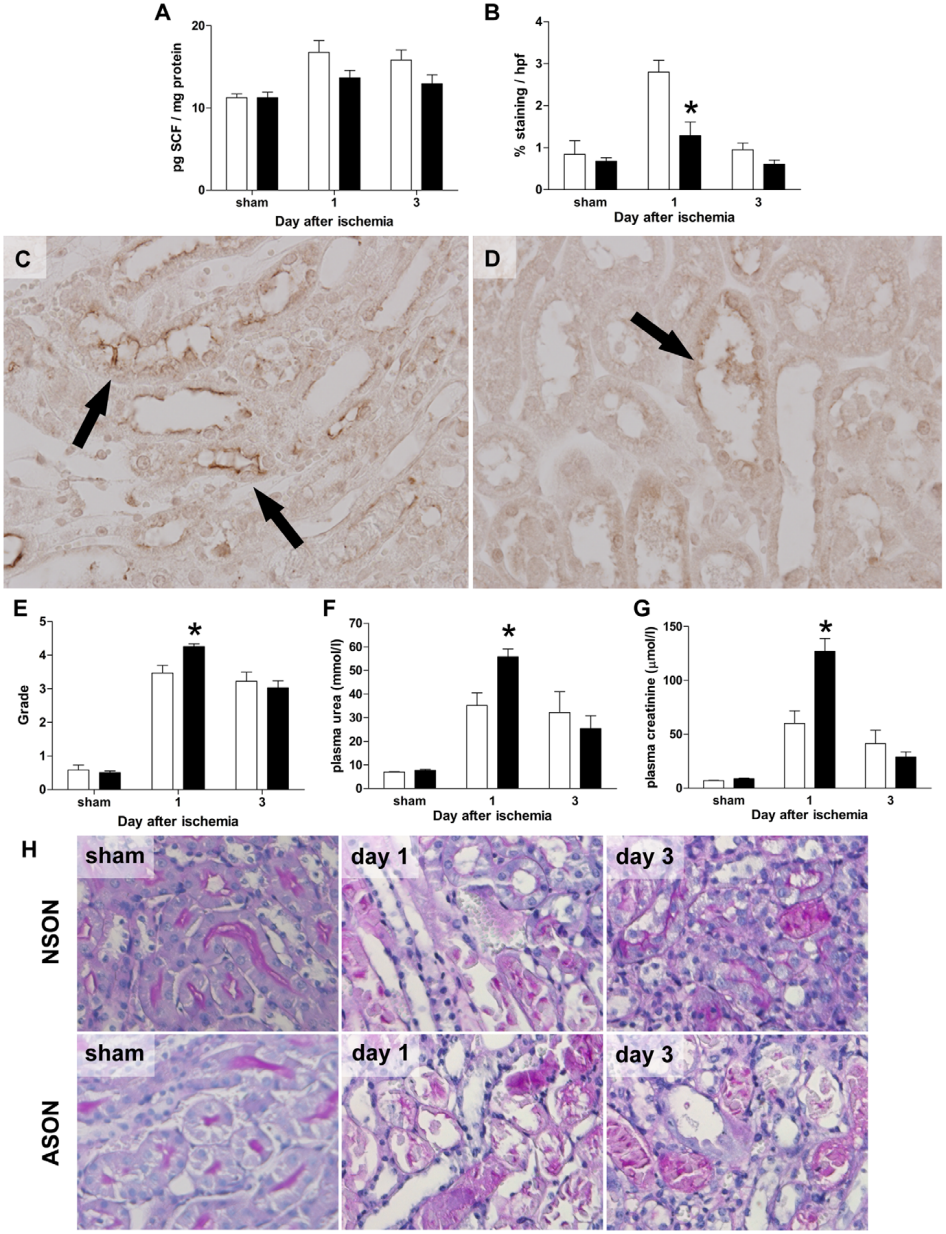
SCF ASON treatment increases renal injury. (A) Whole kidney sample analysis of SCF expression by ELISA did not show any significant difference in SCF levels between NSON (□) and ASON (▪) treated animals (*P* = 0.09, non significant). Data are expressed as mean±SEM. (B) Digital image analysis of SCF immunostainings per hpf of the corticomedullary region demonstrated significantly less staining on tissue sections from ASON treated animals (▪) compared to controls (□) at day 1 after ischemia (*P = 0.003). Data are expressed as mean±SEM. (C) and (D) Representative SCF immunostainings of ischemic kidney sections used for image analysis of NSON (c) and ASON (d) treated animals; original magnification: 40×. (E) Tubular damage was scored in a semi-quantitative fashion. The degree of injury one day after ischemia was significantly increased in ASON treated animals (▪) compared to NSON controls (□) (*P = 0.005). Data are expressed as mean±SEM. (F) and (G) Renal function was determined by measurement of (f) plasma urea and (g) creatinine values. ASON treatment (▪) resulted in significantly elevated plasma urea (*P = 0.0047) and creatinine (*P = 0.001) compared to NSON controls (□) at day one after ischemia. Data are expressed as mean±SEM. (H) Representative PAS-D stainings illustrating tubular injury as scored in (E). Kidneys from sham operated animals after NSON and ASON treatment show no histologic signs of tubular injury. In contrast, at day 1 after ischemia most tubules in the corticomedullary area appear dilated with loss of the brush border or display denudation of the tubular basement membrane. In NSON-treated animals, more undamaged tubules were present compared to ASON-treated animals.

SCF-specific ASON treatment did not lead to a significant decrease of SCF concentrations in whole kidney samples compared to those found in control NSON treated animals subjected to ischemia as measured by ELISA (figure 4A). Since ODN are taken up only by tubules in the corticomedullary area and not by the tubules in the renal papilla, we next examined whether selective down-regulation of SCF expression by proximal tubules after ischemia could be detected by performing digital image analysis of SCF-specific immunostaining in the corticomedullary area of kidney sections. This revealed a significant decrease in the total area stained for SCF in the corticomedulary area of ASON treated animals (figure 4B), suggesting that the antisense treatment did reduce SCF translation during I/R injury specifically in this area of interest compared to NSON controls (figure 4C and D).

PAS-D stainings showed a significantly higher degree of tubular damage in sections of ischemic kidneys of ASON treated animals compared to NSON treated animals (figure 4E). In accordance, renal function was severely impaired in ASON treated animals with significantly higher plasma urea (figure 4F) and creatinine (figure 4G) levels in ASON treated mice compared to control mice at one day after ischemia.

Of importance, in a separate experiment animals were treated with NSON or vehicle only. I/R injury in vehicle treated animals induced rises of plasma urea and creatinine levels that were similar to that found in NSON treated animals, showing no additional renotoxic effect of the phosphorothioate capped oligonucleotides on renal function (data not shown).

### SCF ASON treatment has no effect on renal inflammation but increases TEC apoptosis

**Figure 5:**
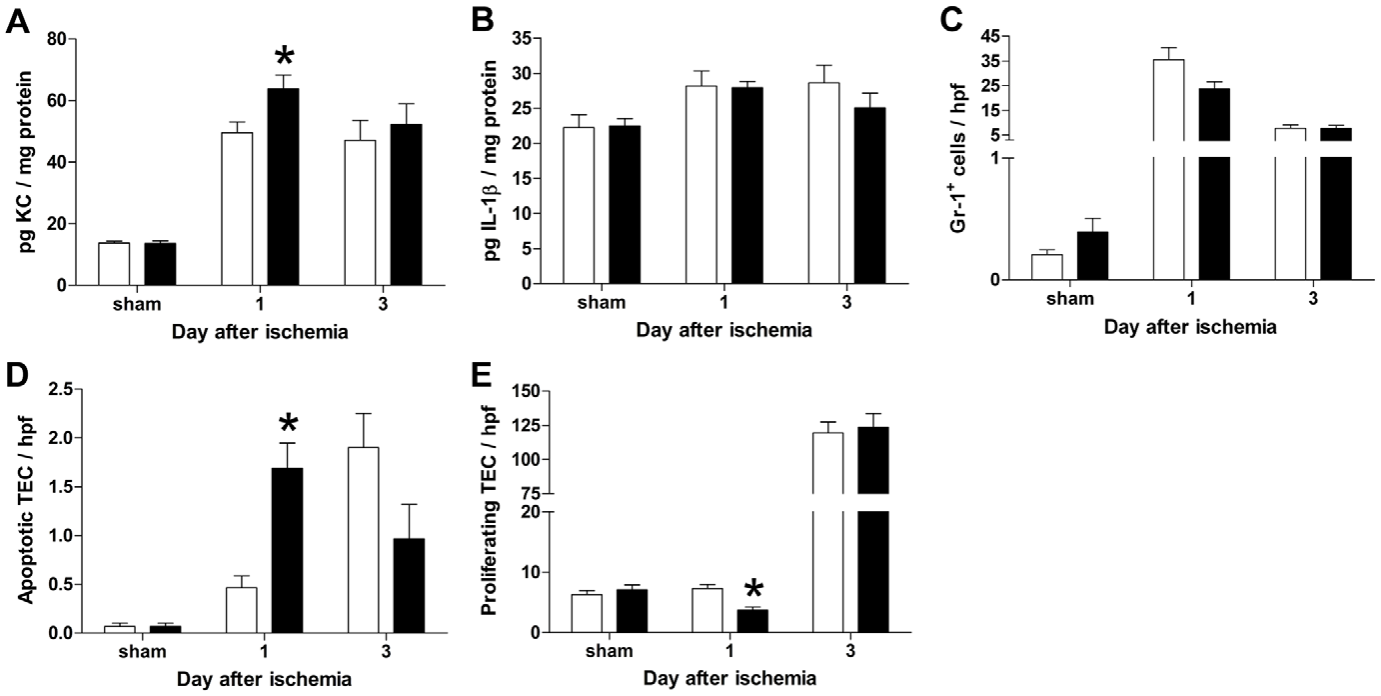
Effect of SCF ASON treatment on inflammation and TEC apoptosis. (A and B) The concentration of renal (A) KC and (B) IL-1β was measured by ELISA in whole kidney homogenates. An increase in the concentration KC was detected in samples obtained from ASON treated animals (▪) subjected to ischemia compared to controls (□) (*P = 0.03). No significant difference was detected in the concentration of IL-1β between NSON (□) and ASON (▪) treated animals. Data are expressed as mean±SEM. (C) Gr-1 positive cells were counted per hpf. Few Gr-1 positive passenger leukocytes were detected in sham operated animals. In contrast, the number of Gr-1 positive cells increased at day 1 after ischemia. No significant statistical difference was detected between the number of positive cells per hpf in NSON (□) or ASON (▪) treated animals. Data are expressed as mean±SEM. (D) Apoptosis of TEC was determined by immunostaining of tissue sections using an antibody to the active form of caspase 3. Positive TEC were counted per hpf, significantly more apoptotic TEC were detected in ASON treated animals (▪) compared to controls (□) at day 1 after ischemia (*P = 0.0005). Data are expressed as mean±SEM. (E) Proliferation of TEC was determined using an antibody to Ki.67 (right). Kidneys from ASON treated animals (▪) contained less proliferating TEC compared to NSON controls (□) at day 1 after ischemia (*P = 0.0009). Data are expressed as mean±SEM.

Early post-ischemic injury is characterized by the influx of granulocytes, being mostly neutrophils, induced by expression of chemotactic factors such as KC/CXCL1. This influx of granulocytes is correlated with increased tubular damage and loss of renal function [2], [25], [26]. Despite the increased tissue damage (figure 4E) and worsening of renal function we observed in SCF ASON treated animals (see figures 4F and G), we noted a similar number of infiltrating granulocytes in the ischemic kidney compared to the NSON treated controls (figure 5C). In contrast, KC/CXCL1 levels were higher in ASON animals (figure 5A), whereas IL-1β levels did not differ between both groups (figure 5B). Analysis of macrophage inflammatory protein 2-alpha (MIP-2α) and monocyte chemotactic protein-1 (MCP-1) protein levels showed that these chemokines were not expressed in different concentrations in renal tissue obtained from ASON-treated and control animals at all time points (data not shown). As ASON treatment also did not increase granulocyte accumulation and that being a decisive factor in renal inflammation, we believe that SCF does not play an important role in inflammation-mediated tissue damage. Next we determined whether decreased SCF expression acts primarily on TEC integrity.

SCF/c-KIT signaling is associated with cell survival and proliferation for different cell types [27]. To determine whether ASON treatment had any effect on TEC apoptosis, we performed stainings for the active form of caspase 3. We detected more apoptotic tubular epithelial cells in ASON treated animals after ischemia compared to controls (figure 5D). In addition, we noted significantly less Ki.67 positive tubular epithelial cells following ischemia in ASON treated animals (figure 5E), suggesting that also TEC proliferation was affected by SCF downregulation. Cell death was confirmed by performing terminal deoxynucleotidyl transferase (TdT)-mediated dUTP nick-end labeling (TUNEL) on renal tissue sections and the number of TUNEL stained cells in the corticomedullary area was determined. TUNEL analysis confirmed the results obtained with the immunostaining for active caspase 3, showing a significant increase in the number of TUNEL stained cells in kidneys from ASON-treated animals at day 1 after ischemia (16.7±0.2 versus 28.9±0.7 for NSON and ASON-treated animals respectively, mean±SEM, *P = 0.01).

### Expression of SCF and c-KIT phosphorylation by IM-PTEC cells after *in vitro* hypoxia

As demonstrated above, SCF exerts protective effects on tubules during I/R injury. To dissect the mode of action of SCF on TEC, we generated and characterized a conditionally immortalized proximal tubular epithelial cell line (IM-PTEC) to investigate how SCF – c-KIT signaling may induce cell survival following renal ischemia using an *in vitro* model.

**Figure 6:**
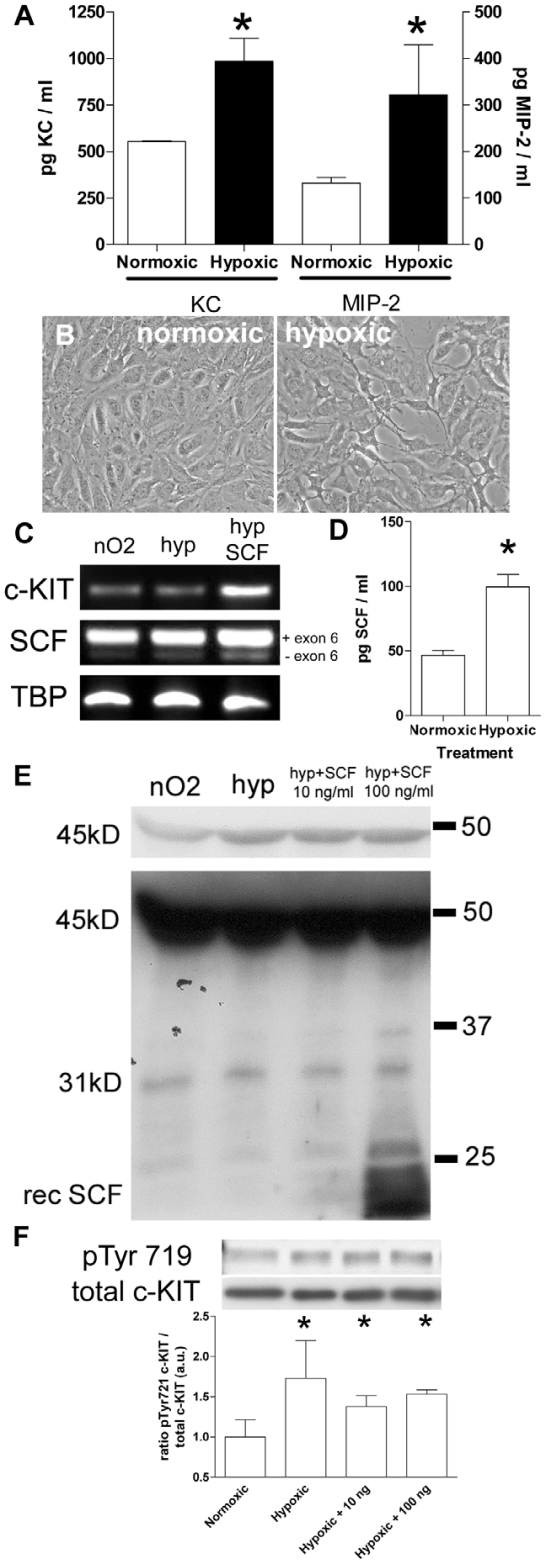
Hypoxia of IM-PTEC results in SCF secretion. (A) To validate the effect of paraffin oil immersion on IM-PTEC, KC and MIP-2 levels were measured in culture medium 24 hrs after hypoxia. In accordance with previous reports, our *in vitro* hypoxia model led to increased KC and MIP-2 secretion by IM-PTEC. Data are expressed as mean±SEM, results are combined of data from two independent experiments (*P = 0.03). (B) Normoxic cells (left) displayed normal epithelial morphology with the appearance of cell-cell contacts. Cell subjected to 60 minutes of hypoxia (right) displayed cellular retraction and loss of cell-cell contacts, indicative of damage by hypoxic stress. Original magnification: 20×. (C) Transcription of c-KIT and SCF was determined in normoxic control cells (nO_2_) and following hypoxia (hyp) and SCF stimulation following hypoxia (100 ng/ml, hyp SCF). Samples were obtained at 24 hours following hypoxia. Dual bands for SCF show the two splice variants designated as full-length Kit Ligand (KL) -1 and KL-2, which lacks exon 6. TATA box binding protein (TBP) was used as a loading control. Data shown here are representative results obtained from 3 separate experiments. (D) Levels of SCF were measured in medium samples from cells subjected to *in vitro* hypoxia using a murine SCF specific ELISA. The concentration SCF obtained from control samples was low and values were just above the detection limit of the ELISA. Hypoxia induced a significant increase in medium SCF. (E) Western blot analysis of conditioned culture medium samples of control cells (nO2), hypoxic cells (hyp), hypoxic cells cultured with 10 ng SCF/ml medium (hyp + SCF 10 ng/ml) or hypoxic cells cultured with 100 ng SCF/ml medium (hyp + SCF 100 ng/ml). Upper panel: 45 kD bands representing full length membrane SCF (KL-1) appear after short exposure. Lower panel: longer exposure reveals a smaller SCF variant approximately 31–32 kD in size and recombinant SCF (approx. 18 kD). Hypoxia leads to increased levels of KL-1 in medium samples whereas the addition of SCF induces increased levels of the smaller SCF forms in the medium. All samples were collected 24 hours following hypoxia. Data shown here are representative results obtained from 3 separate experiments (*P = 0.04). (F) Western blot analysis of cell lysates obtained from cells 24 hours after being subjected to hypoxia. Phosphorylation of tyrosine 719 of c-KIT (pTyr719) was present in samples from normoxic control cells. Hypoxia increases the relative rate of c-KIT phosphorylation. Upper panel: phospho Tyr719 c-KIT. Lower panel: total c-KIT. Densitometric analysis of the relative increase of Tyr719 c-KIT phosphorylation versus total c-KIT following hypoxia. Phosphorylation of Tyr719 phosphorylation in control cells was set as 1. Western blot data shown here are representative results obtained from 3 separate experiments; data from all experiments were used for the densitometric analysis as shown here (*P = 0.05).

To mimic I/R injury *in vitro*, we used a validated model whereby cells are directly immersed in paraffin oil [23], [28]. The resulting hypoxia leads to ATP depletion (not shown), secretion of KC and macrophage inflammatory protein-2 (MIP-2) (figure 6A) and disruption of cell-cell contacts (figure 6B).

Transcription of c-KIT and both SCF splice variants was active in normoxic controls and cells subjected to hypoxia followed by a 24 hour recovery while maintained in medium with or without recombinant rat (rr) SCF (100 ng/ml) (figure 6C). Since SCF exists both as a membrane bound and soluble protein, we measured SCF in medium samples by ELISA. Hypoxia resulted in a two-fold increase of soluble SCF compared to controls (figure 6D). To determine which SCF variants are present in medium samples of cells subjected to in vitro hypoxia, we performed Western blot analyses on samples. Interestingly, we readily detected the presence of the 45 kD KL-1 SCF variant in medium samples obtained from control cells. The intensity of the respective bands seemed to increase in samples from cells subjected to hypoxia (figure 6E, upper panel). The smaller KL-2 isoform and the cleaved, soluble form of KL-1 have a size of 32 and 31 kD, respectively. We detected a band at longer exposure times corresponding with a protein of approximately that size which does not appear to increase in intensity following hypoxia (figure 6E, lower panel).

To determine whether signaling via c-KIT is involved in the cellular response to hypoxia, we studied if phosphorylation of the tyrosine residue at position 719 (Tyr719) occurs, since phosphorylation of this residue is engaged in activation of multiple signaling pathways [7]. Control normoxic cells maintained in culture medium already displayed phosphorylation of Tyr719, but this was increased in cells subjected to hypoxia and left to recover for 24 hours in culture medium supplemented with 10 or 100 ng/ml SCF or vehicle (figure 6F).

### SCF reduces apoptosis of tubular epithelial cells and induces phosphorylation of Bad

**Figure 7:**
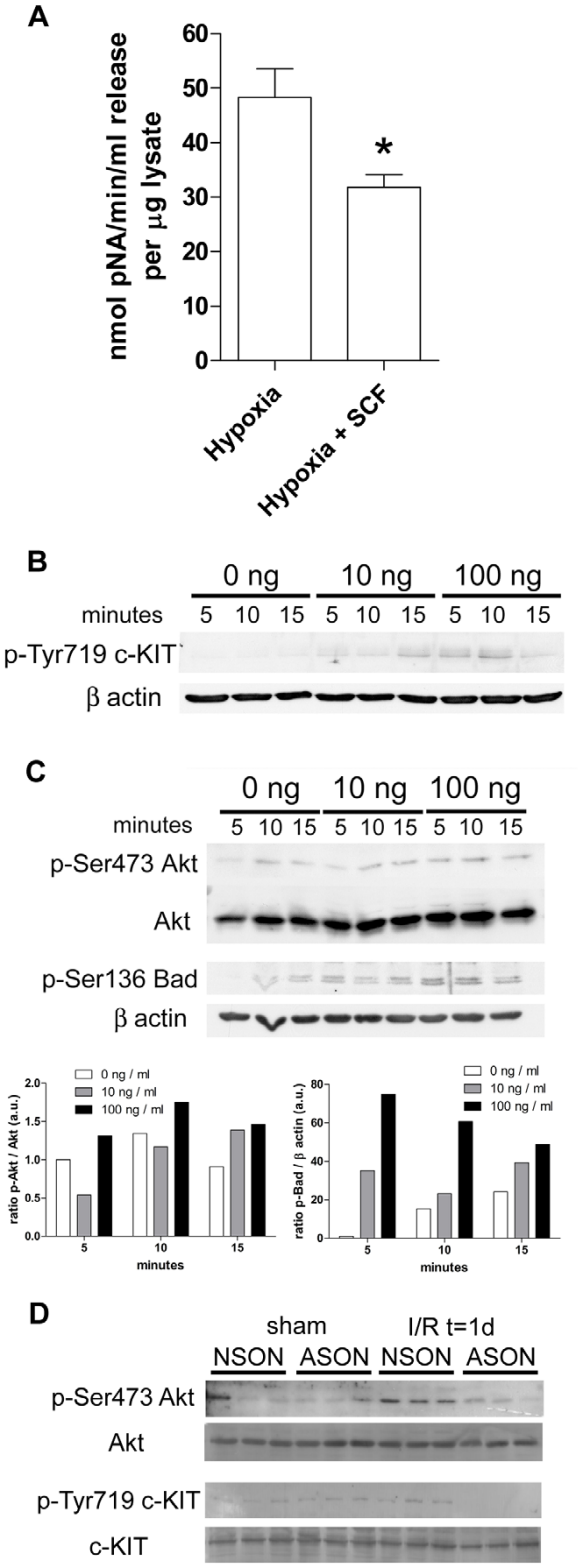
SCF reduces hypoxia induced IM-PTEC apoptosis and induces phosphorylation of Bad. (A) SCF reduces caspase 3 activity following hypoxia. IM-PTEC cells were subjected to in vitro hypoxia and cultured with 0 ng or 100 ng SCF/ml medium for 24 hours. Cell lysates were collected and assayed for caspase 3 activity. To correct for equal input, values are expressed as activity per µg protein. SCF reduces caspase 3 activity in IM-PTEC cells following in vitro hypoxia (*P = 0.026). Data are expressed as mean±SEM. Results are obtained from 2 separate experiments with 6 measurements each. (B) IM-PTEC cells were serum starved and exposed to 0, 10 or 100 ng SCF/ml for 5, 10 and 15 minutes. Protein samples were analyzed using Western blot. SCF induced phosphorylation of Tyr719 of c-KIT; β actin was used as loading control. (C) SCF results in phosphorylation of Ser473 of Akt; total Akt expression was used as loading control (upper panel); SCF induces phosphorylation of Ser136 of Bad; β actin was used as loading control (lower panel). Normalized densitometric analyses of the immunoblots are presented as the ratio phosphorylated protein/loading control. Data shown here are representative results obtained from 3 separate experiments. (D) Renal tissue samples obtained from sham and animals subjected to ischemia were analyzed for phosphorylation of Akt (p-Ser473 Akt) and c-KIT (p-Tyr719 c-KIT). Expression of total Akt and c-KIT were used as loading control.

Apoptosis in I/R injury is associated with increased caspase 3 activity [29]. From pilot experiments we determined that at 24 hours after hypoxia, caspase 3 activity was significantly increased compared to controls (data not shown). We measured this activity in IM-PTEC cells subjected to hypoxia and cultured for 24 hours with or without 100 ng/ml SCF during the subsequent reoxygenation. Hypoxia led to a significant increase in the activity of caspase 3, but addition of SCF significantly reduced this activity (figure 7A).

To determine the mechanism of SCF induced inhibition of apoptosis, we stimulated cells with both 10 ng/ml or 100 ng/ml rrSCF and examined protein phosphorylation of downstream signaling pathways in c-KIT/SCF signaling. We focused on pathways which are activated by SCF induced phosphorylation of Tyr719 on c-KIT, which is essential for PI_3_K signaling [7]. Addition of SCF resulted in rapidly increased phosphorylation of Tyr719 (figure 7B) compared to non-stimulated cells. In addition, phosphorylation of Akt (figure 7C, upper panels) and Bad (figure 7C, lower panels), both downstream of PI_3_K, occurred upon SCF stimulation. This suggests that cell survival in IM-PTEC cells by SCF is mediated by phosphorylation of the tyrosine residue at position 719 on c-KIT, leading to subsequent phosphorylation of Akt and Bad resulting in decreased apoptosis. Interestingly, no differences in phosphorylation of p38, p42/44 and JNK MAPK between stimulated and control cells were observed (data not shown).

To correlate these findings to our *in vivo* data, we analyzed phosphorylation of Akt and c-KIT in renal homogenates from animals treated with NSON and ASON (figure 7D) All samples from NSON-treated animals after ischemia showed increased phosphorylation of Akt compared to samples from sham-operated animals. ASON-treatment reduced phosphorylation of Akt after ischemia compared to NSON-treated controls. In samples from sham-treated animals, a low level of c-KIT phosphorylation could be detected which was similar to that in samples from NSON-treated animals that had been subjected to renal ischemia. No specific signal was detected in lanes loaded with samples from ASON-treated animals, demonstrating reduced activation of c-KIT and Akt after ASON-mediated down-regulation of SCF expression.

## Discussion

Expression of c-KIT is generally regarded as a marker for adult stem cells or their more differentiated descendants. Examples of tissue stem cells expressing c-KIT are the well-known hematopoietic stem cell [30], [31], cells from the testes [12] and the recently identified cardiac progenitor [32]. However it is incorrect to attribute stem cell-like properties to any cell expressing c-KIT. Mast cells [9], cells of Cajal in the intestines [33], vascular smooth muscle cells [9] and epithelial cells from the distal nephrons [19], [20], for example, also express c-KIT, without there being evidence that these cells posses any stem cell-like properties. No distinctly shared phenotypes between c-KIT expressing cells have been observed, suggesting that c-KIT expression does not indicate cellular multipotency *per se*. Here we observed expression of c-KIT by TEC in the corticomedullary area of the kidney following ischemia, but not in normal kidneys.

During the early reperfusion phase following renal ischemia, extensive necrosis and apoptosis takes place of the tubular epithelium located in the corticomedullary area of the kidney. It is the loss of the tubular epithelium in this region, comprising the proximal tubular compartment that correlates with loss of renal function as can be detected by increased urea and creatinine levels in the blood. Viable, surviving TEC have been implicated in tubular epithelial restoration [5] and their presence may be essential in the balance between repair and permanent loss of renal tissue. A role for cytokine mediated TEC protection following acute renal failure as caused by ischemia has been described previously for erythropoietin (EPO) [34]. In agreement with our findings, EPO is expressed following hypoxia and protects the tubular epithelium against cell death. However, EPO expression was limited to interstitial fibroblasts, whereas its receptor was expressed on TEC [34]. Both c-KIT as its ligand SCF were present in damaged tubules, suggesting that this interaction might represent a protective, autocrine cell survival mechanism.

Apoptosis of TEC is an important determinant of renal function in I/R injury. TEC apoptosis following ischemia may be the result of various stimuli; however all routes converge at the point of caspase 3 activation as a common end point [29]. Here we found that SCF leads to phosphorylation of Bad via activation of Akt, which is known to prevent apoptosis, as has previously been demonstrated [35]. Decreased SCF expression resulted in increased TEC apoptosis as determined by detection of active caspase 3 *in vivo*, whereas addition of SCF to hypoxic IM-PTEC *in vitro* significantly decreased caspase 3 activity. The mechanism underlying the protective role of SCF may thus result from Bad phosphorylation leading to suppression of caspase 3 activity. It is previously reported that SCF can mediate cell-matrix adhesion of mast cells [36] and may induce Slug transcription [37]. SCF may thus also increase TEC adhesion following injury which may add to the anti-apoptotic properties of SCF. We did not observe any effect of SCF on MAPK phosphorylation. With respect to p38 and p42/44 MAPK activation, membrane bound SCF will induce a more persistent signal, compared to soluble SCF [7], which may partially explain no increased phosphorylation upon ligand stimulation. Activation of p38 and JNK MAPK have been shown to facilitate apoptosis of TEC following injury [38]. This observation therefore underlines our hypothesis that SCF protects tubules to ischemic injury via the SCF/c-KIT signaling pathway which prevents apoptosis of TEC.

Han *et al*. [39], have previously demonstrated that hypoxia is able to induce SCF expression in mammary carcinoma cells via binding of hypoxia-inducible factor 1α (HIF-1α) to a hypoxia-response element located in the promoter region of SCF. Epidermal growth factor (EGF) was shown to increase SCF expression further. These findings may provide a mechanism for the sharp increase in SCF expression at day 1 after ischemia. Further studies should demonstrate whether HIF-1α and EGF are the driving force behind activation of SCF expression in the proximal tubules during I/R injury.

Local down-regulation of SCF in the corticomedullary area did not affect granulocyte influx following ischemia when compared to control animals. As tubular injury, tubular epithelial proliferation and apoptosis were affected by ASON-treatment, we investigated the role of SCF/c-KIT signaling using an *in vitro* model for hypoxia. In line with this, we found a role for SCF in cell survival following *in vitro* hypoxic injury. Furthermore, we could establish that SCF induces phosphorylation of c-KIT and Bad, suggesting that this pathway is involved the survival of cells following *in vitro* hypoxia. Using kidney lysates from NSON and ASON-treated animals, we found that c-KIT phosphorylation was virtually absent after ischemia in ASON-treated animals but not in the NSON-treated controls. This was reflected by increased phosphorylation of Akt in control animals after ischemia which was lower in ASON-treated animals.

Several SCF knockout animals have been described whereby most homozygote SCF mutations are lethal due to severe anemia [40]. Mice that are compound heterozygotes for the SCF alleles Kitl^Sl^/Kitl^Sl-d^ are viable, but display severe defects such as macrocytic anemia but also renal malformations. These include thickening of the glomerular basement membrane, increased glomerular cellularity [41] but also increased mesangial matrix deposition and severe malformations of the distal nephrons (personal observation). This phenotype does not permit the use of these animals in experimental renal I/R injury. We have therefore applied a different strategy to block SCF expression by preventing mRNA translation using ASON treatment. This approach has several important benefits over other approaches. First, expression is only transiently reduced and bypasses the occurrence of adaptive mechanism that may be observed in knockout animals as result of the specific genetic deletion. Second, phosphorothioate capped oligonucleotides are distributed to the kidney [35], more specifically to the glomerular parietal and the tubular epithelium in the corticomedullary area [23], [24] and has been used in previous studies with success. The fact that we found no differences between vehicle and NSON treated animals with respect to tubular injury or renal function following I/R injury indicates that the oligonucleotides do not affect TEC by inducing renoprotection or, the opposite, being cytotoxic. Unfortunately we are unable to demonstrate the effect of ASON on translation of target genes *in vitro*. We speculate that upon *in vitro* cell culture, the proximal TEC lose their capacity to properly engage in reabsorption or uptake processes as a result of imperfect polarization, thus limiting the uptake of oligonucleotides. However, addition of SCF to hypoxic cells *in vitro* does supplement and support our *in vivo* findings by decreasing the rate of apoptosis in cultured IM-PTEC cells whereas a decrease of SCF expression by ASON treatment increases apoptosis of TEC *in vivo*.

Here we have shown that c-KIT and SCF expression occurs in tubules in the corticomedullary area during I/R injury. Reduced expression of SCF leads to increased TEC apoptosis. Hypoxia has been shown to regulate SCF expression *in vitro* and addition of SCF reduces caspase 3-mediated apoptosis via phosphorylation of Bad. This protective interaction appears to be an autocrine mode of TEC survival following I/R injury. Whether SCF and c-KIT also mediate other protective adaptations to hypoxic injury *in vivo* and *in vitro* has to be determined in future studies.

## Materials and Methods

### Mice, I/R injury, antisense treatment and oligonucleotide distribution

Eight to 10 week old male C57BL/6 mice (B6) and Immorto mice were purchased from Charles River (Maastricht, The Netherlands). Bilateral ischemia was induced for in B6 mice as described previously [2]. In short, mice were anesthetized by an intraperitoneal injection with 0.08 mg/ml fentanyl-citrate, 2.5 mg/ml fluanison (Janssen Pharmaceuticals, Beerse, Belgium) and 1.25 mg/ml midazolam (Roche, Mijdrecht, The Netherlands). Renal pedicles were exposed following a midline incision. Both renal arteries were clamped for 45 minutes using microaneurysm clamps during which the incision was provisionally closed. After removal of the clamps, reperfusion was confirmed by visual inspection. The abdomen was irrigated with 100 µl of saline (0.9% w/v) and the incision was closed using non-absorbable sutures (Tyco Healthcare, Gosport, UK). All mice received a subcutaneous injection of 50 µg/kg buprenorphin (Temgesic; Schering-Plough) for analgetic purposes and were allowed to recover from surgery for 12 hours at 32°C in a ventilated stove. Animals were sacrificed at day 1, 3, 7 and 14 after ischemia (n = 10 per time point) to analyze SCF expression. Sham operated animals (n = 8) were handled similarly with the exception of clamping of the renal pedicles and sacrificed at day 1 after ischemia. Phosphorothioate capped antisense (ASON) specific to mouse SCF RNA and control nonsense (NSON) oligonucleotides were obtained from Biognostik (Göttingen, Germany). ASON and NSON were dissolved in sterile saline and administered in a volume of 100 µl containing 4 nmol oligonucleotides intraperitoneally at the day prior to ischemia and directly into the abdominal cavity after release of the clamps. Animals (for I/R: n = 10 per group, for sham: n = 6 per group) were sacrificed at day 1 after ischemia.

To analyze oligonucleotide tissue distribution, FITC-labeled NSON were administered intraperitoneally twice with an interval of 24 hours between injections. Animals were sacrificed five hours after the last administration.

Plasma samples were obtained by blood collection via heart puncture using a 25 gauge needle. Blood was transferred to lithium-heparin gel tubes (BD Microtainer, BD, Breda, The Netherlands) and stored on ice until processing. Plasma was obtained after centrifugation of tubes in a standard table centrifuge (10,000 rpm, 10 minutes, 4°C) as supernatant. Kidneys were excised after which the renal capsule was removed. Kidneys were divided transversally and processed as described below. All experimental procedures were approved by the Animal Care and Use Committee of the University of Amsterdam, the Netherlands (study number DPA1008). Experiments have been conducted according to the national guidelines.

### Antibodies

The following antibodies were used for immunostainings and immunoblotting: biotinylated rat-anti-mouse c-KIT from R&D (Abingdon, UK), rat-anti-mouse Gr-1 (BD Biosciences, Alphen a/d Rijn, The Netherlands), rabbit-anti-Ki.67, rabbit-anti- fluorescein isothiocyanate (FITC) from DAKO (Glostrup, Denmark), rabbit-anti-activated caspase 3, anti-phospho-Akt, anti-phospho p38 mitogen-activated protein kinase (MAPK), anti-phospho p42/44 MAPK, anti-phospho-c-Jun N terminal kinase (JNK), anti-JNK, anti-phospho-tyrosine(Tyr)721 c-KIT and anti-phospho-BAD from Cell Signaling (Danvers, MA, USA), rabbit-anti-Akt, rabbit-anti-ERK1/2, goat-anti-c-KIT and goat-anti-phospho-Tyr721 c-KIT from Santa Cruz (Santa Cruz, CA, USA), rabbit-anti-mouse-SCF from Chemicon (Chandlers Ford, UK). The anti-β actin antibody was from Sigma-Aldrich (St Louis, MO). The anti-CD10 from Neomarkers (Runcorn, UK) and anti-aquaporin 4 from Millipore (Amsterdam, The Netherlands). Secondary peroxidase (HRP), alkaline phosphatase (AP), FITC or Texas Red (TxR) conjugated antibodies were all from DAKO.

### (Immune) Histochemistry (IHC) and TUNEL staining

Kidneys were fixed in formalin for approximately 10 hrs or snap frozen in liquid nitrogen. Formalin fixed tissues were subsequently embedded in paraffin overnight in a routine fashion. Fourµm thick sections were cut and used for staining with periodic acid Schiff reagents after diastase digestion (PAS-D). For immunostainings, all antibodies were diluted in PBS. Formalin fixed tissue sections were dewaxed and incubated in methanol containing 0.3% H_2_O_2_ for 15 minutes. Sections were subsequently boiled for 10 minutes in 10 mM citrate (pH 6.0) prior to SCF or goat-anti-c-KIT immunoglobulin or in 10 mM Tris/1 mM EDTA (pH 9.0) prior to caspase 3 antibody labeling. To detect Gr-1, sections were digested with 0.25% pepsin (Sigma) dissolved in 0.1 M HCl for 15 minutes at 37°C. Sections were blocked for 30 minutes in PBS containing 5% normal goat serum (Jackson Immunoresearch, Newmarket, UK). Sections were incubated with primary antibodies for 2 hours, HRP-conjugated secondary antibodies for 30 minutes, both at room temperature. Sections were stained using 3,3′-diaminobenzidine and alternatively counterstained with haematoxylin. For double immunostainings, cells were dewaxed and boiled in citrate buffer as described above. All antibodies for double stainings were diluted in Antibody Diluent (Immunologic, Duiven, The Netherlands).Tissue was blocked with V-Block (Immunologic) and incubated simultaneously with CD10 or SCF specific antibodies overnight, followed by labeling with a CD10 or SCF specific AP-conjugated secondary antibody for 30 minutes and staining was performed using Vector Blue (Vector Laboratories). Sections were retreated with citrate as above, blocked 5% normal swine serum (Jackson) and labeled for 3 hours with c-KIT specific antibody followed by labeling with a AP-conjugated secondary antibody. Staining was performed using Vector Red (Vector Laboratories).

For immunofluoresence stainings, 10 µm thick cryosections were fixed in cold acetone for 15 minutes. TxR labeled phalloidin (Molecular Probes) was dissolved as described in the product information. Sections were treated with 0.1% Triton X-100 in PBS for 10 minutes, rinsed in PBS, and incubated for 5 minutes with phalloidin. For fluorescence stainings, sections were treated with 0.1% azide in PBS for 15 minutes, incubated with biotin block (DAKO) as described in the product information and blocked for 15 minutes using serum-free block (DAKO). Sections were incubated with primary antibodies for 2 hours, FITC and TxR-conjugated secondary antibodies for 30 minutes, both at room temperature. Sections were mounted with Vectashield (Vector Laboratories, Peterborough, UK) containing 4′,6-diamidino-2-phenylindole (DAPI).

Terminal deoxynucleotidyl transferase (TdT)-mediated dUTP nick-end labeling (TUNEL) was performed on paraffin embedded tissue sections using the fluorescein-based In Situ Cell Death Detection kit (Roche) according to the instructions of the manufacturer. In short, tissue sections were dewaxed followed by a 15 minute pre-treatment with Proteinase K (Roche, 5 µg/ml in 10 mM Tris/HCl, pH 7.4) at 37°C followed by a PBS wash. After labeling, sections were washed in PBS and labeled for 20 minutes with rabbit-α-FITC IgG (Dako), washed with PBS and labeled with goat-α-rabbit-poly-alkaline phosphatase (Immunologic) for 30 minutes. Staining was performed using Vector Red.

### Triple immunostaining and spectral imaging

For triple immunostaining, all sections were treated as above and both epitope retrieval and immunolabeling for each antigen were performed in a consecutive order. Sections were counterstained with eosin and mounted. Single immunostainings were performed for adjustment of spectral imaging wave length settings per chromogen. Light microscopy images were acquired with a LeicaDM6000B microscope (Leica, Wetzlar, Germany) and specific stainings were unmixed using Nuance software (CRi, Woburn, MA).

### Histopathological scoring and renal function

Damage to tubular epithelium was assessed using PAS-D stainings, all histopathological scorings were performed in the corticomedullary area on 10 non-overlapping fields (at 400× magnification) and performed in a blinded fashion. Damage was graded and scored semi-quantitatively on a scale from 0–5 [2], [23].

Renal function was determined by measurement of plasma creatinine and urea concentrations using Crea-plus (Roche Diagnostics, Almere, The Netherlands) in a routine fashion at the clinical diagnostics department of our institute.

### TEC isolation and generation of a conditionally immortalized PTEC cell lines

TEC were isolated from Immorto mice as described previously [23], labeled with antibodies to neprilsyin/CD10 and aquaporin 4 combinedly, as markers for proximal tubular epithelium [42], [43] and sorted by flow cytometry on a FacsAria cell sorter (BD Biosciences). Cells were grown in HK-2 medium (DMEM/F12 medium (Invitrogen) with 5% fetal bovine serum (Hyclone, Etten-Leur, The Netherlands), 5 µg/ml insulin and transferrin, 5 ng/ml sodium selenite (Roche), 20 ng/m tri-iodo-thyrionine (Sigma), 50 ng/ml hydrocortisone (Sigma) and 5 ng/ml prostaglandin E1 (Sigma) with L-glutamine and antibiotics (both from Invitrogen, Paisley, UK) and mouse interferon-γ (IFN-γ, 1 ng/ml, R&D)) at 33°C in 5% CO2 and 95% air. From this cell population, monoclonal cell lines were generated by limiting dilution and examined for downregulation of SV40 activity during restrictive conditions by immunofluoresence (data not shown) with recurrence of the cobble stone-like morphology (data not shown). One clone was used for all experiments described below and named IM-PTEC hereafter. Cells were grown in flasks until 70% confluent and then passed to the appropriate assay plates. All experiments were performed with cells between passage 4 and 15 after start of the culture. Cells were seeded in 6 well assay plates at a density of 25,000 cells per well and cultured for 2 days under permissive cell culture conditions reaching approximately 50% confluence. Cells were then washed with PBS and cultured for one week under restrictive culture conditions.

### In vitro hypoxia

Hypoxia was simulated as described previously [23], [28]. In short, IM-PTEC cells were briefly serum-starved for 2 hrs in DMEM/F12 medium. After washing, cells were incubated for 60 minutes with mineral paraffin oil (BUFA, Uitgeest, The Netherlands), controls with serum free medium. ATP depletion was confirmed by measuring intracellular ATP levels in cell lysates using the Bioluminescence Assay Kit HS II (Roche) as described previously [23] (data not shown). Cells were washed twice with serum free medium, and reoxygenation was permitted for 24 hrs after hypoxia in HK-2 medium. Recombinant rat-SCF was purchased from Peprotech (Rocky Hill, NJ, USA). Medium was removed and stored for further analysis; cells were washed with PBS and processed for immunoblotting or reverse transcription PCR.

### SCF stimulation

Prior to stimulation, cells were serum starved for 2 hrs. SCF (10 or 100 ng/ml medium) or vehicle (controls) was added to the cells and incubated at 37°C for 5, 10 or 15 minutes. Stimulation was stopped by washing cells in ice cold PBS. Cells were lysed as described below.

### In vitro cell apoptosis assay

Apoptosis was measured using the Caspase 3 Assay Kit (Sigma) according to the manufacturer's protocol. Cell medium containing detached cells was transferred to tubes and placed on ice, adherent cells were washed with PBS, trypsinized and added to the proper medium samples. Cells pellets were washed with PBS once and lysed using the provided buffer. Samples were stored at −80°C until caspase 3 activity measurement. Lysates from cells grown in HK2 medium were used as negative controls.

### Transcription analyses reverse transcription PCR (RT-PCR)

Cells and cryosection of renal tissue were lysed with TRIZOL to isolate RNA. RT-PCR was performed with 2.5 units/sample of Taq DNA polymerase (Invitrogen) using 35 cycles and an annealing temperature of 58°C. Primers were designed as follows. SCF primers for RT-PCR were designed to span exon 6 to demonstrate both splice variants. All primers were synthesized by Sigma-Genosys (Cambridgeshire, UK).

For RT-PCR:

c-KIT: 5′-ATCCCGACTTTGTCAGATGG-3′ and 5′-CGTCTCCTGGCGTTCATAAT-3′

SCF: 5′-GGGATGGATGTTTTGCCTAGT-3′ and 5′-GTCCATTGTAGGCCCGAGT-3′

TATA binding protein (TBP): 5′-CAGGAGCCAAGAGTGAAGAAC-3′ and 5′-GGAAATAATTCTGGCTCATAGCTACT-3′

### ELISA and Western blotting

Frozen kidneys were thawed on ice for 30 minutes in PBS supplemented with 4 mM EDTA, 1% Triton X-100 containing relevant protease inhibitors (Sigma), and subsequently homogenized using a PT1300D disperser (Kinematic AG, Lucerne, Switzerland) at 30,000 rpm for 10 seconds. Duoset ELISA kits specific for detection of mouse KC, IL-1β, MIP-2α and MCP-1 were obtained from R&D and performed in accordance to the supplied protocol. Antibody dilutions were used as specified in the accompanying datasheet. In short, standard ELISA assay plates (Corning) were incubated overnight with chemokine specific capture antibody, blocked with 1% (w/v) BSA in PBS for one hour and incubated with tissue homogenate (one volume homogenate diluted in one volume blocking solution for all chemokines except KC which was diluted in 4 volumes of blocking solution) for 2 hours. Detection antibody was incubated for 2 hours, followed by labeling with the provided HRP-conjugated streptavidin. The mouse SCF ELISA kit was purchased from Peprotech and performed in accordance with the supplied protocol. All antibodies were used at dilutions described in the data sheet. Detection antibody, streptavidin-HRP and tissue homogenates (1∶1) were diluted in diluent buffer (0.1% (w/v) BSA, 0.05% (v/v) Tween-20 in PBS). Further procedures were similar to that of other ELISA sets. All ELISA stainings were developed using *o*-Phenylenediamine dihydrochloride (0.4 mg/ml, Sigma) dissolved in citrate buffer (7.3 g sodium citrate, 11.9 g Na_2_HPO_4_⋅2H_2_O per 500 ml, pH 5.6). Development was stopped by addition of 1 M H_2_SO_4_. Absorbance was measured at 490 nm. Cytokine levels were corrected for total protein content per sample using a Bradford protein assay (Bio-rad, Veenendaal, The Netherlands).

For immunoblotting, cells and kidney tissue were lysed in RIPA buffer (50 mM Tris (pH 7.5), 0.15 M NaCl, 2 mM EDTA, 1% (w/v) deoxycholic acid, 1% (v/v) nonidet P40 (Roche), 0.1% (w/v) sodium dodecyl sulfate) supplemented with protease inhibitor cocktail (Sigma, diluted 100-fold), 10 mM NaF and 4 mM Na_3_VO_4_. Protein concentrations were determined using the bichonic acid protein assay kit (BCA; Thermo Fisher Scientific, Rockford, IL). One volume of samplebuffer (0.5 M Tris-HCl (pH = 6.8), 20% glycerol (v/v), 10% β mercaptoethanol (v/v), 4% SDS (w/v), 0.005% bromophenolblue) was added to five volumes of lysate and heated to 100°C for 5′. Samples were separated on 7.5% (c-KIT), 15% (Bad, SCF) or 10% (other proteins) acrylamide gels and blotted on PVDF membranes (Millipore, Leiden, The Netherlands) for 2 hours at 100 V. Immunoblots for cell lysates were blocked in 5% (w/v) non-fat dry milk in Tris buffered saline with 0.1% Tween 20 (Sigma) (TBS-T) for use with all non-phospho-specific antibodies and with 5% (w/v) BSA in TBS-T for all phospho-specific antibodies for 1 hour. Immunoblots for kidney samples were blocked in 0.2% (w/v) I-Block (Applied Biosystems, Nieuwerkerk a/d IJssel, The Netherlands) in TBS-T for 1 hour. Antibodies were diluted in the respective blocking buffer and blots were incubated O/N at 4°C. Secondary HRP- or AP-conjugated antibodies were diluted 1/5000 in the respective blocking buffer and incubated with blots for 30–60 minutes. Detection of HRP-conjugated antibodies (used with cell samples) was performed using ECL+ (Amersham) and imaged with a Typhoon 9400 imager (GE Healthcare, Diegem, Belgium) or ECL (Thermo Fisher Scientific) using autoradiography films (GE Healthcare). AP-conjugated antibodies (used with kidney samples) were detected using the Tropix Western-Star Alkaline Phosphatase kit (Applied Biosystems) using autoradiography films (GE Healthcare). Densitometric analyses were performed using ImageJ (National Institutes of Health, USA, URL: http://rsb.info.nih.gov/ij/index.html).

### Statistical analyses

Results are expressed as mean ± standard error of the mean (SEM). Data were first tested for normality using the Kolmogorov-Smirnow test and then analyzed using an unpaired *t* test. Tubular injury scores were analyzed using the non-parametric Mann-Whitney U Test. Values of *P*≤0.05 were considered statistically significant. All statistical analyses were performed using Graphpad Prism4 (GraphPad Software, San Diego, CA, USA).
